# Human dental pulp stem cell adhesion and detachment in polycaprolactone electrospun scaffolds under direct perfusion

**DOI:** 10.1590/1414-431X20186754

**Published:** 2018-03-26

**Authors:** A. Paim, D.I. Braghirolli, N.S.M. Cardozo, P. Pranke, I.C. Tessaro

**Affiliations:** 1 Laboratório de Separação por Membranas, Departamento de Engenharia Química, Universidade Federal do Rio Grande do Sul, Porto Alegre, RS, Brasil; 2 Laboratório de Simulação, Departamento de Engenharia Química, Universidade Federal do Rio Grande do Sul, Porto Alegre, RS, Brasil; 3 Laboratório de Hematologia e Células-Tronco, Faculdade de Farmácia, Universidade Federal do Rio Grande do Sul, Porto Alegre, RS, Brasil; 4 Instituto de Pesquisa com Células-Tronco, Porto Alegre, RS, Brasil

**Keywords:** Cell adhesion, Perfusion, Shear stress, Stem cell, Electrospun scaffolds

## Abstract

Cell adhesion in three-dimensional scaffolds plays a key role in tissue development. However, stem cell behavior in electrospun scaffolds under perfusion is not fully understood. Thus, an investigation was made on the effect of flow rate and shear stress, adhesion time, and seeding density under direct perfusion in polycaprolactone electrospun scaffolds on human dental pulp stem cell detachment. Polycaprolactone scaffolds were electrospun using a solvent mixture of chloroform and methanol. The viable cell number was determined at each tested condition. Cell morphology was analyzed by confocal microscopy after various incubation times for static cell adhesion with a high seeding density. Scanning electron microscopy images were obtained before and after perfusion for the highest flow rate tested. The wall pore shear stress was calculated for all tested flow rates (0.005–3 mL/min). An inversely proportional relationship between adhesion time with cell detachment under perfusion was observed. Lower flow rates and lower seeding densities reduced the drag of cells by shear stress. However, there was an operational limit for the lowest flow rate that can be used without compromising cell viability, indicating that a flow rate of 0.05 mL/min might be more suitable for the tested cell culture in electrospun scaffolds under direct perfusion.

## Introduction

In tissue engineering, scaffolds are used as substitutes for damaged tissue and act as a support for cell proliferation, differentiation, and migration. In order to promote the formation of natural extracellular-matrix, a scaffold must be designed with appropriate biocompatibility, biodegradability, architecture, and mechanical properties (1).

An important class of scaffolds for tissue engineering is based on electrospun polymer-based structures comprising solid microfibers or nanofibers, which can present high packing density and interconnected pore network (2). Nanofiber scaffolds favor higher mesenchymal stem cell viability than smooth surfaces (3). However, nanofiber scaffolds usually present small pores (4) that can hinder cell infiltration through three-dimensional structures (2). On the other hand, microfiber scaffolds can provide structures with bigger pores, allowing the cell migration and colonization inside the matrix (5).

Perfusion culture systems enhance mass transfer in scaffold-containing bioreactors and provide increased nutrient transport and cell viability (6), migration (7), growth, and differentiation (8). In addition, perfusion bioreactors can reduce the accumulation of toxic metabolites and degradation byproducts and the polymer degradation rate (9). Nevertheless, high shear stress can provoke cell detachment followed by cell death (10). Consequently, the cell number in three-dimensional (3D) scaffolds under perfusion is influenced by the cell detachment provoked by shear stress (11) and the capability of the cells to remain adhered to the scaffold and to proliferate, differentiate, and migrate is strongly dependent on the flow rate and the pore size employed. This is important because in order to obtain a homogeneous and effective regeneration of damaged tissue, it is essential to produce a biomaterial with an adequate cell number for implantation. Therefore, it is necessary to quantify the cell drag and the final cell number in perfusion bioreactors to produce tissue substitutes that fit the quality standard required in a medical environment. Despite this, many studies on perfusion systems based on 3D scaffolds focus on the flow rate and shear stress effect on nutrient transport and stem cell proliferation and differentiation (12–14), without evaluating the cell detachment from the scaffold.

This work addressed the reduction of the shear stress effects inside the scaffold pores under perfusion to produce cellularized electrospun structures for clinical application. An investigation was made of flow rate and shear stress under direct perfusion in polycaprolactone electrospun scaffolds on human dental pulp stem cell detachment. The influence of the adhesion time on cell adhesion and detachment under static conditions was also evaluated. Different seeding densities were tested under perfusion to evaluate the detachment.

## Material and Methods

### Scaffold production

The scaffolds were produced in an electrospinning apparatus with temperature and humidity control (EC-CLI, IME Technologies, Netherlands). A 16% w/w solution of polycaprolactone (Sigma-Aldrich, USA; Mw 70–90 kDa) in a chloroform:methanol 9:1 vol% mixture was electrospun at 38% humidity, 19°C, 35 cm distance between the needle and the collector, flow rate of 0.1 mL/min, and voltage of 17 kV. The scaffolds were cut into 16 mm diameter disks and sterilized by ultraviolet radiation (UV) for 1 h.

### Cell isolation and expansion

The pulp of human deciduous teeth was used to obtain dental pulp stem cells with the approval of the Research Committee and the Ethics Committee of the Universidade Federal do Rio Grande do Sul (project No. 33177214.1.3001.5330), according to the methodology described by Werle et al. (15). Human deciduous teeth with physiologic root resorption were extracted and immersed in DMEM (Dulbecco’s modified Eagle’s culture medium)/Hepes (Sigma-Aldrich), supplemented with 10% fetal bovine serum (FBS; Gibco, USA), 100 U/mL penicillin and 100 mg/mL streptomycin (Gibco), for transportation. The dental pulp tissue was removed with the use of endodontic instruments and the cells were isolated from the pulp by a mechanic and enzymatic process. The isolated cells were incubated for 24 h at 37°C and 5% CO_2_. The primary cultures and further passages were subcultured when a confluence of 90% was reached, with medium exchange every 3 or 4 days. Five primary culture cells (between the third and eighth passages) were used in this work. The cells were characterized as mesenchymal stem cells in terms of immunophenotypic profile and differentiation potential, as presented in the Supplementary Material (Table S1 and Figure S1, respectively).

### Cell viability

A colorimetric assay with water-soluble tetrazolium salts (WST-8, [2-(2-methoxy-4-nitrophenyl)-3-(4-nitrophenyl)-5-(2,4-disulfophenyl)-2H-tetrazolium]) was used to determine the number of viable cells. As opposed to other common colorimetric assays, the WST-8 assay does not kill the cells, which allows continuous culturing of the cells, performing other assays with the living cells, and preserving the samples after the measurements. For this test, the culture medium was removed and the scaffolds were incubated with 20 µL of Cell Counting Kit-8 solution (CCK-8, Sigma-Aldrich) and 180 µL of fresh culture medium at 5% CO_2_ and 37°C for 1 h. The absorbance was read in 450 nm in a microplate reader (Multiskan FC, Thermo Scientific, USA). Standard curves relating absorbance readings (450 nm) with cell number were constructed for each cell culture to determine the viable cell number.

### Cell morphology

After the viability assay, the scaffolds were washed with phosphate-buffered saline solution (PBS) and fixed with 4% (w/v) paraformaldehyde (PFA; Sigma-Aldrich) for 30 min. The PFA was then removed and the scaffolds were washed again with PBS. Cytoskeleton and nuclei cells were stained with 50 µg/mL rhodamine-phalloidin (Molecular Probes, USA) for 40 min and 0.5 µg/mL 4′,6-diamidino-2-phenylindole (DAPI) for 5 min, respectively, for further imaging using a confocal microscope (Olympus FV1000, Japan). For scanning electron microscopy (SEM; JEOL JSM 6060, Japan), the scaffolds were washed with PBS, fixed with 3% glutaraldehyde and dehydrated in graded ethanol baths before being sputtered with gold and imaged.

### Scaffold properties

The porosity of scaffolds was calculated from the volume of fibers and the total volume of the scaffold. The volume of the fibers (V_fibers_) of the scaffold was determined by dividing the weight of the scaffold by the PCL density, and the total volume of the scaffold (V_total_) was determined taking into account its geometry. The total porous fraction (θ), thereby, is given by Equation 1.

$$\theta =\frac{V_{total}-V_{fibers}}{V_{total}}$$ (Eq. 1)

The average shear stress on the wall of the pores was calculated considering a cylindrical pore approximation (16), using Equation 2.

$$\sigma =\frac{8\mu Q}{d_{p}\theta \pi {\left(\frac{D}{2}\right)}^{2}}$$ (2)

where μ is the medium viscosity (Pa.s) (similar to that of water at 37°C), d_p_ is the pore diameter (m) (measured through analysis of SEM images of the scaffolds with the software ImageJ), Q is the flow rate (m^3^/s), and D is the scaffold diameter (m).

### Cell adhesion and drag

Initially, 1.5×10^5^ or 0.5×10^5^ cells were seeded in each scaffold and the volume was completed to 1 mL with culture medium in each well. Different adhesion times - 3 h (17), 6 h (18), and 24 h (19,20) - were used for mesenchymal stem cell attachment in the 3D scaffolds. In order to evaluate the impact of the adhesion time on cell adhesion and morphology, cells were then incubated at 5% CO_2_ and 37°C for 3, 6, and 24 h for cell adhesion. Three cell cultures were used in this experiment and the number of scaffolds analyzed for each adhesion time was 3 per culture.

**Figure 1.**
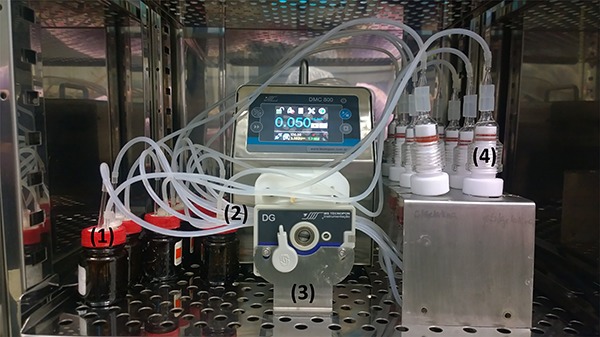
Perfusion bioreactor system: medium reservoir (1), silicon tubing (2), peristaltic pump (3), and perfusion chamber (4).

Firstly, the scaffolds seeded with 0.5×10^5^ cells and incubated for 3, 6, and 24 h were perfused with culture medium for 18 h at 0.05 mL/min, in order to evaluate the adhesion strength of each adhesion time group. Two scaffolds per culture were analyzed for each adhesion time under flow and three cell cultures were used. The bioreactor system used for the culture medium perfusion is shown in Figure 1.

Considering that increased cell adhesion and spreading have been reported in the literature with longer adhesion times (19–21), the effect of the flow rate in cell detachment was evaluated following an adhesion time of 24 h under static conditions, to guarantee that the cells were fully adhered and would not be detached with flow perfusion due to poor adhesion. In addition, the perfusion time used in further experiments was changed from 18 to 24 h to guarantee that maximum cell drag was achieved. Therefore, the scaffolds with 24 h cell adhesion were transferred to the bioreactor chambers (Figure 1) and incubated at 5% CO_2_ and 37°C for 24 h of perfusion in a bioreactor system.

**Figure 2.**
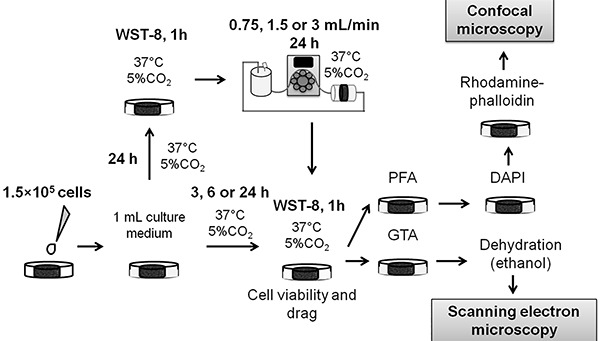
Scheme of the experimental procedure for cell culture with seeding density of 1.5×10^5^ cells/scaffold (WST-8, PFA, GTA, and DAPI are, respectively, the viability assay, paraformaldehyde, glutaraldehyde, and 4′,6-diamidino-2-phenylindole).

**Figure 3.**
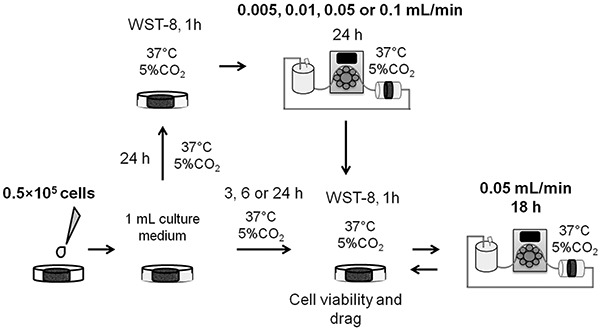
Scheme of the experimental procedure for cell culture with seeding density of 0.5×10^5^ cells/scaffold (WST-8 is the viability assay).

As cell drag was expected to increase with the flow rate, the scaffolds with high and low cell densities were perfused at high and low flow rates, respectively, to guarantee that the viability measurements remained above the detection limit. Thus, the scaffolds seeded with 0.5×10^5^ cells were perfused with culture medium at the flow rates of 0.005, 0.01, 0.05, and 0.1 mL/min (Figure 2), while those seeded with 1.5×10^5^ cells were perfused at 0.75, 1.5, and 3 mL/min (Figure 3). Two cell cultures were used in these experiments and the number of scaffolds analyzed for each flow rate was 4 per culture.

The schemes of the experimental procedure for cell adhesion and perfusion experiments are presented in Figures 2 and 3, with an indication of the steps in which cell viability and morphological characteristics were determined.

It was also considered that with the reduction of the flow rate, the time required to fill the perfusion system increased. Hence, if the arrival of culture medium to the scaffold occurs with an increased delay, the cells could starve due to the lack of nutrients. Thus, at low flow rates (0.005–0.1 mL/min), the perfusion chambers were at first partially filled with culture medium at a higher flow rate (0.5 mL/min). The flow rate was then decreased to 0.005, 0.01, 0.05, or 0.1 mL/min before the fluid reached the scaffold in any of the chambers.

The average drag ratio was calculated by subtracting the average cell number (determined with the CCK-8 kit) after perfusion from the control average cell number (non-perfused scaffold).

### Statistical analysis

Normal distribution was verified with the combination of the Shapiro-Wilk test and visual inspection of Q-Q plots. Statistical analyses were performed using one-way ANOVA followed by Tukey's *post hoc* test, and were carried out with R Statistical Software (version 3.3.2; R Foundation for Statistical Computing, Austria).

## Results and Discussion

### Cell morphology

**Figure 4.**
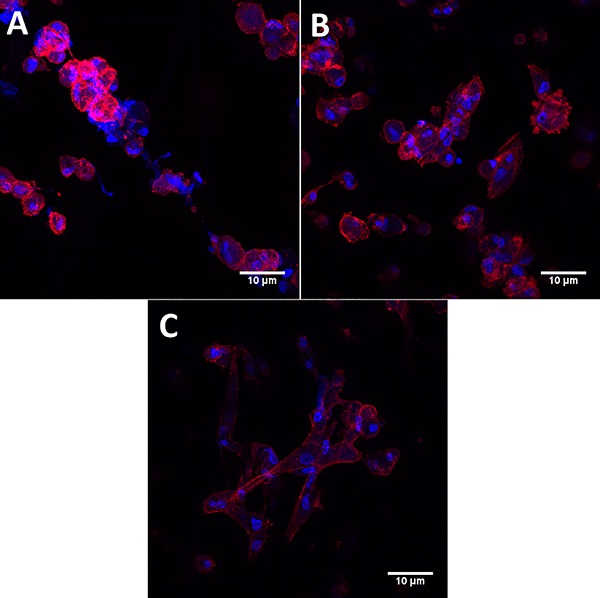
Confocal images of stem cells from culture I in scaffolds seeded with 1.5×10^5^ cells and stained with rhodamine-phalloidin (cell cytoskeleton in red) and DAPI (cell nuclei in blue) after 3 (*A*), 6 (*B*), and 24 h (*C*) cell adhesion. Magnification ×40.

Figure 4 presents the confocal images of scaffolds seeded with 1.5×10^5^ cells and incubated for 3, 6, and 24 h. Additionally, a similar set of images with smaller magnification can be seen as Supplementary Material (Figure S2) to show that the effects observed in Figure 4 do not depend on the specifically focused region. It can be observed that the cell shape was still round after 3 h of adhesion (Figure 4A). At 6 h (Figure 4B), the area of actin fibers stained with phalloidin was higher and after 24 h of adhesion, a spread morphology can be observed (Figure 4C). These results indicate that cytoskeleton spreading was increased with longer adhesion times. As larger cell spreading has been associated with increased focal adhesion size (22) and strength (23), it can be expected that after 24-h adhesion, the cells will be more strongly attached to the fibers of the scaffold.

**Figure 5.**
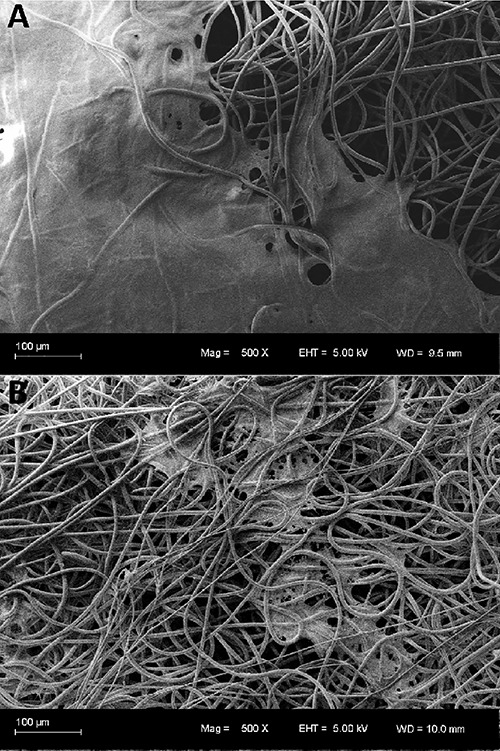
SEM images of scaffolds seeded with 1.5×10^5^ cells for 24 h with cell culture V before (*A*) and after (*B*) culture medium perfusion for 24 h with a flow rate of 1.5 mL/min. Magnification ×500.

To evaluate the cell distribution on the scaffold surface, SEM analyses were performed before and after perfusion. Figure 5 presents SEM images of the scaffolds seeded with 1.5×10^5^ cells after 24-h cell adhesion followed by 24-h perfusion. As can be seen, after 24-h adhesion, the cells organize themselves in agglomerates, forming regions containing a layer of cells on the matrix surface, obstructing the pores. Several studies support that the pore size is responsible for cell infiltration and shear stress is applied to the cells due to the passage of flow in perfusion bioreactors (24,25). In this study, the porosity (93±1%) and pore diameter of the scaffolds (11.97±4.36 µm) did not necessarily limit cell penetration into the scaffold, if the cell diameter range of 12.2–16.6 μm is considered, as suggested by Suchanek et al. (26). However, with the static seeding, the cells adhered on the fibers covering the scaffold surface and occupying the pore spaces (as shown in Figure 5A). Due to fluid flow, the cells obstructing large pores were detached and dragged with the culture medium under high velocities (Figure 5B), and eventually detached because of the shear stress applied, even under low flow rates.

### Cell adhesion and drag

**Figure 6.**
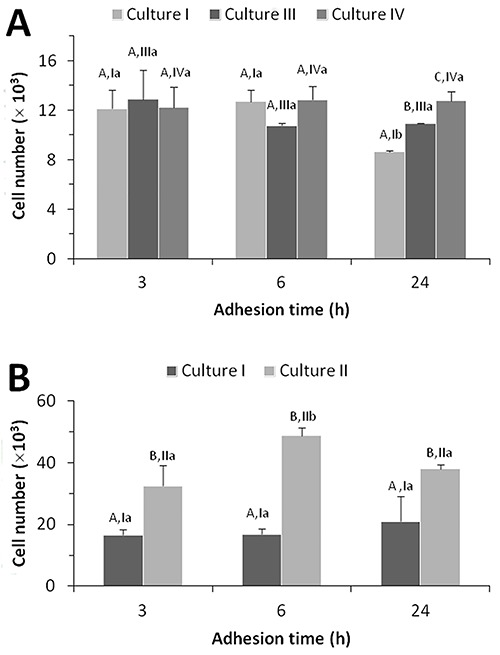
Cell number for different adhesion times for cell cultures I, III, and IV seeded with 0.5×10^5^ cells (*A*) and for cell cultures I and II seeded with 1.5×10^5^ cells (*B*). Data are reported as means±SD. Different capital letters represent significantly different means for the groups with the same adhesion time. Different lowercase letters preceded by the culture number represent significantly different means for the groups of the same culture with different adhesion times (one-way ANOVA with *post hoc* Tukey test, P<0.05).

Figure 6 presents the results of cell number, determined by WST-8, for the adhesion times of 3, 6, and 24 h for the scaffolds seeded with a low seeding density (0.5×10^5^ cells/scaffold) with the cell cultures I, III, and IV (Figure 6A) and with a high seeding density (1.5×10^5^ cells/scaffold) with the cell cultures I and II (Figure 6B). For low seeding density, there was no significant effect of adhesion time on cell number determined by cell viability for cultures III and IV, while for culture I a significant (P<0.05) decrease of mean cell number occurred after 24-h adhesion compared to the lower adhesion times (3 and 6 h). For high seeding density, there was no significant difference between the values of the mean cell number obtained at the different adhesion times for culture I. For culture II, a decrease of this parameter was also observed between 6 and 24 h, after an initial increase between the adhesion times of 3 and 6 h. However, these results are probably exhibiting a behavior related to the intrinsic characteristics of this specific cell culture, which can be an outcome with primary cell cultures due to the variability between donors (27–31). Harumi Miyagi et al. (31) observed donor-to-donor variation of the expression of extracellular matrix proteins with different human dental pulp stem cells from deciduous teeth, which could justify the different adhesion behavior between the cultures presented in Figure 6. This is in agreement with the fact that mesenchymal stem cells, as anchorage-dependent cells, can undergo cell death by the lack of appropriated attachment to a substrate (32).

A further aspect to be mentioned about Figure 6 is that at both low and high seeding density, significant differences between the cultures regarding the number of cells were observed. This can be a result of the use of cells derived from different individuals. Donor-to-donor variability can occur due to several factors such as donor age and gender, and it has been reported in several studies with primary cultures of human mesenchymal stem cells (27–31).

**Figure 7.**
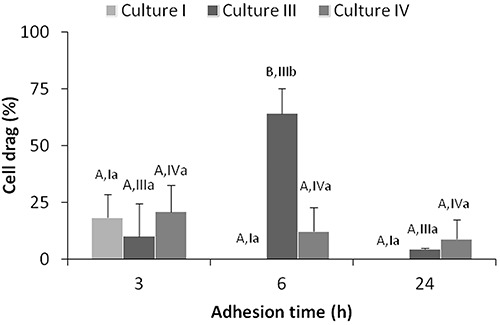
Cell drag in scaffolds seeded with 0.5×10^5^ cells with different adhesion times and perfused with a flow rate of 0.05 mL/min (shear stress of 2.1 mPa) for 18 h. Data are reported as means±SD. Different capital letters represent significantly different means for the groups with the same adhesion time. Different lowercase letters preceded by the culture number represent significantly different means for the groups of the same culture with different adhesion times (one-way ANOVA with *post hoc* Tukey test, P<0.05).

Figure 7 presents the cell drag percentage calculated from the viable cell numbers (determined by WST-8) obtained for the scaffolds seeded with 0.5×10^5^ cells and perfused at a flow rate of 0.05 mL/min for 18 h. As can be seen, there was no effect of adhesion time in cell loss under perfusion at 0.05 mL/min for cultures I and IV because no significant difference was observed for the different adhesion time groups. In addition, mean cell drag, calculated as the average drag from the three cultures, presented no significant difference between the different adhesion time groups (mean cell drag of 17±11, 20±28, and 5±6% for scaffolds with 3, 6, and 24 h of adhesion time, respectively). However, culture III presented significantly different cell drag when seeded with 6-h adhesion compared to the other cultures with the same adhesion time (P<0.001), and to the same culture with other adhesion times (P<0.001). Furthermore, culture I presented no cell loss for 6 and 24 h (0% cell drag). These reduced cell losses can be related to a higher cell spreading observed at 6 and 24 h of adhesion, observed in Figure 4. Similar results to those obtained for cultures I and IV were observed by van Kooten et al. (33) in bi-dimensional studies using parallel-plate flow chambers, where tangential flow was used to induce shear stress and detach a cell population from a surface. The authors observed that cell adhesion strength, determined as the shear stress level that promotes 50% of cell detachment, was not sensitive to adhesion time. However, 3D attachment results in different cell morphology (bridged form) than cell adhesion in 2D structures (flat shape) (34). Furthermore, reduced cell adhesion strength and resistance to shear stress can be observed in 3D scaffolds under perfusion conditions because the cells can adhere in an orientation normal for the flow and lead to increased cell detachment under low flow rates (35). However, cell attachment in bi-dimensional structures result in flat form morphology (34). In this study, with the increase of adhesion time, the cells, initially adhered to the fibers (Figure 4A), stretched through the fibers and the pore space to adhere to other cells and fibers (Figure 4C). This is in accordance with the bridged form morphology (cells attached to more than one fiber) obtained by Binulal et al. (34) for cell attachment in 3D electrospun scaffolds. Furthermore, since the cells adhered filling the pore space (Figure 5), the main orientation to cell attachment in the studied system is expected to be perpendicular to the flow direction, which differs from the parallel flow used by van Kooten et al. (33). According to McCoy and O’Brien (35), reduced cell adhesion strength and resistance to shear stress can be observed in 3D scaffolds under perfusion conditions, because cells can adhere in an orientation normal for the flow and lead to increased cell detachment under low flow rates. This could explain the mechanism of cell drag in a direct perfusion system and the distribution of the cells after perfusion, observed in Figure 5B. Thus, it could be that cultures I and IV presented no enhancement in adhesion strength, with larger adhesion times due to this relationship between cell morphology and flow direction.

**Figure 8.**
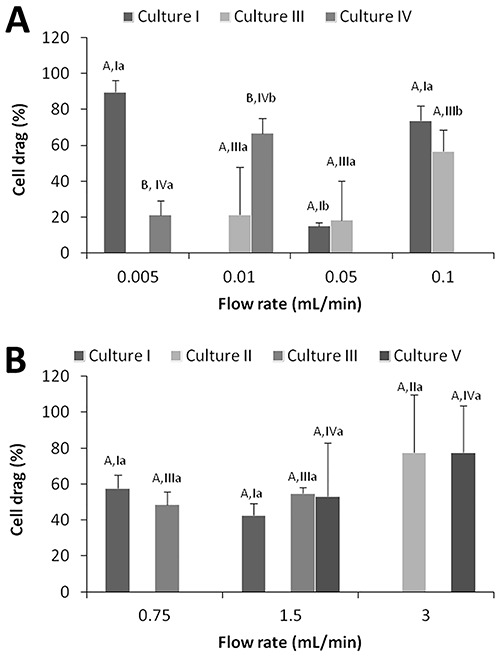
Cell drag percentage in scaffolds seeded with 0.5×10^5^ cells from cell cultures I, III, and IV, perfused with flow rates varying from 0.005 to 0.1 mL/min (*A*), and in scaffolds seeded with 1.5×10^5^ cells from cultures I, II, III, and V, perfused with flow rates from 0.75 to 3 mL/min (*B*), with 24 h of adhesion and perfused for 24 h. Data are reported as means±SD. Different capital letters represent significantly different means for the groups with the same flow rate. Different lowercase letters preceded by the culture number represent significantly different means for the groups of the same culture with different flow rates (one-way ANOVA with *post hoc* Tukey test, P<0.05).

Figure 8 presents the values of cell drag percentage calculated from viable cell numbers (determined with WST-8) in the scaffolds from cell cultures I, III, and IV, seeded with 0.5×10^5^ cells and perfused with flow rates varying from 0.005 to 0.1 mL/min (Figure 8A) and from cultures I, II, III, and V, seeded with 1.5×10^5^ cells and perfused with flow rates from 0.75 to 3 mL/min (Figure 8B). In all cases, the cell drag percentages were determined after 24-h adhesion followed by 24-h perfusion. As can be seen, at low seeding density, higher flow rates led to a significant increase in cell loss for culture IV (P<0.001). This was also observed for the scaffolds seeded (0.5×10^5^ cells) with cultures I and III (P<0.01) compared to the results with flow rates of 0.05 and 0.1 mL/min. On the other hand, culture I, when seeded at a low density, presented higher cell loss for the flow rate of 0.005 mL/min compared to the flow rate of 0.05 mL/min (P<0.0001). For very low flow rates as 0.005 mL/min, the loss of cell viability (observed for culture I) is probably not provoked by shear stress but by the reduction of oxygen delivery inside the perfusion chamber, because of the decreased convection. Decreased oxygen concentrations have already been reported with reduced convection in perfusion bioreactors (36,37). Furthermore, the cell drag differences observed between the cultures can be a result of the use of cells derived from different individuals, as previously mentioned. Interestingly, at higher flow rates and seeding density there was no significant difference in cell loss between the different groups with different flow rates and/or cultures (Figure 8B). This can be due to the higher seeding density, which results in higher cell number at the beginning of the perfusion and in an initial reduction of the permeability of the scaffold due to superficial pore obstruction. With less free space for fluid flow, the pore diameter and porosity of the scaffold are reduced, increasing shear stress levels (as in accordance with Equation 2) and cell drag. Additionally, it was observed that as cells are detached by the passage of flow through the pores, the amount of cells and debris in suspension is increased (results not shown). It is possible that, with a high flow rate, the high quantity of cellular particles in suspension affected the viscosity of the culture medium, also increasing the shear stress levels (as in accordance with Equation 2). The combination of these factors with the variability in seeding efficiency between the cultures with high seeding density (observed in Figure 6B) could have homogenized the cell drag with different flow rates.

Table 1 shows the values of mean cell drag percentage and shear stress (Equation 2) obtained with the scaffolds seeded with 0.5 and 1.5×10^5^ cells for 24-h adhesion and perfused with flow rates from 0.005 to 3 mL/min for 24 h. The lowest cell drag (24%) was obtained with a flow rate of 0.05 mL/min, which results in a shear stress of 2.1 mPa on the pore walls. This result indicates that there is an optimal flow rate value for each system, providing this flow rate does not provoke cell starvation or high cell detachment. However, no significant difference was observed for mean cell drag with different flow rates, which could be due to the high standard deviation of each group and donor-to-donor variability. Fibrous (but not electrospun) scaffolds with fiber diameters of 20 µm presented cell detachment values (62 and 69%) at flow rates of 0.5 and 1 mL/min (38) similar to those presented in Table 1 at flow rates of 0.75 and 1.5 mL/min. On the other hand, high shear stresses (values up to 56 and 57 mPa), which are close to those calculated in this work for a flow rate of 1.5 mL/min, have been shown to provoke cell washout and apoptosis (10,39). This could justify the high cell loss presented in Table 1 at high flow rates, as cell drag was calculated based on cell viability, being the possible reason for not observing the general trend reported in direct perfusion systems (i.e., increase in cell detachment with the increase of flow rate) (40) in the present work.

**Table 1.** Mean cell drag and shear stress in scaffolds seeded with 0.5 ×10^5^ and 1.5×10^5^ cells with 24 h of cell adhesion and perfused for 24 h.

	0.5 × 10^5^ cells				1.5 × 10^5^ cells		
Flow rate (mL/min)	0.005	0.01	0.05	0.1	0.75	1.5	3.0
Shear stress (mPa)	0.2	0.40	2.10	4.1	30.9	61.9	123.7
Mean cell drag (%)	63	44	24	65	54	50	78
Standard deviation (%)	35	30	29	13	9	18	26
Cultures	I, IV	III, IV	I, III	I, III	I, III	I, III, V	II, V

The detachment of human dental pulp stem cells from polycaprolactone electrospun scaffolds under direct perfusion was studied for different flow rates, adhesion times and seeding densities. Higher adhesion time led to higher cell spreading in static conditions and reduced cell detachment under perfusion. The seeding density affected cell distribution on the scaffold surface and the sensitivity of the cells to the flow rate. High shear stress and flow rate values resulted in high cell detachments, but too low flow rates were closer to operational constraints that could result in loss of cell viability. Thus, the lowest flow rate within a safe operating range might be more suitable for the culture of human dental pulp stem cells in electrospun scaffolds.

## Supplementary material

Click here to view [pdf].
